# Comparing biological information contained in mRNA and non-coding RNAs for classification of lung cancer patients

**DOI:** 10.1186/s12885-019-6338-1

**Published:** 2019-12-03

**Authors:** Johannes Smolander, Alexey Stupnikov, Galina Glazko, Matthias Dehmer, Frank Emmert-Streib

**Affiliations:** 1 Predictive Society and Data Analytics Lab, Faculty of Information Technology and Communication Sciences, Tampere University, Tampere, Finland; 2 Turku Centre for Biotechnology, University of Turku, Turku, Finland; 3 Department of Oncology, School of Medicine, Johns Hopkins University, Baltimore, USA; 4 Department of Biomedical Informatics, University of Arkansas for Medical Sciences, Little Rock, USA; 5 Institute for Intelligent Production, Faculty for Management, University of Applied Sciences Upper Austria, Steyr, Austria; 6 Department of Mechatronics and Biomedical Computer Science, UMIT, Hall in Tyrol, Austria; 7 College of Artificial Intelligence, Nankai University, China, Tianjin, China; 8 Institute of Biosciences and Medical Technology, Tampere, Finland

**Keywords:** Deep learning, Deep belief network, Classification, Non-coding RNA, Lung cancer and Machine learning

## Abstract

**Background:**

Deciphering the meaning of the human DNA is an outstanding goal which would revolutionize medicine and our way for treating diseases. In recent years, non-coding RNAs have attracted much attention and shown to be functional in part. Yet the importance of these RNAs especially for higher biological functions remains under investigation.

**Methods:**

In this paper, we analyze RNA-seq data, including non-coding and protein coding RNAs, from lung adenocarcinoma patients, a histologic subtype of non-small-cell lung cancer, with deep learning neural networks and other state-of-the-art classification methods. The purpose of our paper is three-fold. First, we compare the classification performance of different versions of deep belief networks with SVMs, decision trees and random forests. Second, we compare the classification capabilities of protein coding and non-coding RNAs. Third, we study the influence of feature selection on the classification performance.

**Results:**

As a result, we find that deep belief networks perform at least competitively to other state-of-the-art classifiers. Second, data from non-coding RNAs perform better than coding RNAs across a number of different classification methods. This demonstrates the equivalence of predictive information as captured by non-coding RNAs compared to protein coding RNAs, conventionally used in computational diagnostics tasks. Third, we find that feature selection has in general a negative effect on the classification performance which means that unfiltered data with all features give the best classification results.

**Conclusions:**

Our study is the first to use ncRNAs beyond miRNAs for the computational classification of cancer and for performing a direct comparison of the classification capabilities of protein coding RNAs and non-coding RNAs.

## Background

Lung cancer is one of the most common cancers in humans worldwide among both men and women, as well as the leading cause of cancer-related deaths [1]. There are two major types of lung cancer, non-small-cell lung cancer (NSCLC) and small-cell lung cancer and adenocarcinoma is a subtype of NSCLC and the most common type in patients who never smoked [1]. In recent years, next-generation sequencing (NGS) technologies have opened an experimental door for the systematic study of complex diseases, including lung cancer, by allowing the generation of high-throughput data on all relevant molecular levels [2, 3]. However, one problem we are facing in this context, is that we are still discovering new variables that might be of crucial importance in understanding the organizational principles of the molecular machinery. For this reason is it not surprising that molecular and genomic medicine are still at its infancy [4–6]. One example for such players are non-coding RNAs (ncRNAs) [7–9].

Non-coding RNAs (ncRNAs) is a broad class of transcripts, consisting of well known transcripts with structural (rRNAs, tRNAs, snRNAs, snoRNAs, etc.) and regulatory (miRMAs, siRNA, piRNAs, etc.) roles, and transcripts whose functions remain largely unknown [10–12]. The latter includes sense/antisense transcripts, ranging in length from 200 bp to 100 kb. Collectively they are called long non-coding RNAs (lncRNAs) (Wang et al., 2011) and sometimes referred to as genomic ’dark matter’ [13, 14]. Large-scale evolutionary properties of the bulk of lncRNAs [15] and the existence of hundreds of experimentally characterized lncRNAs [16, 17] suggest that many of them have a well-defined biological function [12, 13]. The catalogue of the functionally annotated part of the non-coding transcriptome (70-90% of transcribed matter [10, 18, 19] is constantly growing, however, at the moment the total number of non-coding RNAs is unknown. Recent estimates suggest that there are thousands in the human genome [20, 21].

Among the many categories of ncRNAs the most well understood are miRNAs (also called microRNAs), siRNAs and piRNAs, which guide effector Argonaute proteins to genomic loci or target RNAs in a sequence-specific manner. lncRNAs, on the other hand, are implicated mostly in the regulation of gene expression and many are functionally validated by now to be involved in different cellular and developmental pathways [22, 23]. Dysregulation of lncRNAs is observed in many human diseases, including colon cancer, breast cancer, leukemia, ischaemic heart disease, Alzheimer’s disease and some others (see [20] for a review). The ever-growing experimental evidence implicating ncRNAs regulatory roles in many biological processes has led to the idea of using ncRNAs as disease biomarkers, e.g., for diagnostic purposes [24].

Regarding the classification of disorders, lncRNAs have been used to distinguish different subtypes in human breast cancer and glioblastoma [25, 26]. However, all of these studies used unsupervised hierarchical clustering or similar methodology for obtaining the class predictions rather than automized supervised methods [27].

Our paper contributes to these diagnostic investigations by studying the classification capabilities of protein coding and non-coding RNAs from lung cancer. Specifically, we are using RNA-seq data from lung adenocarcinoma patients [28] generated with an Illumina HiSeq 2000 platform containing information about patients with lung cancer and matched control samples from adjacent normal tissue. The availability of RNA-seq data allows us to obtain information about protein coding RNAs and non-coding RNAs. We utilize this opportunity investigating the predictive abilities of both data sources by studying the classification of the lung cancer patients.

We perform this analysis for a number of different state-of-the-art classification methods, including deep learning neural networks, decision trees, random forrests and support vector machines [29–34]. We study the dependency of these methods on a multitude of model parameters, e.g., the neural network architecture, the learning algorithms or the kernels. In addition, we study the influence of feature selection on the results. Our results will shed light on the discriminatory information content of ncRNAs in comparison with coding RNAs.

For our classification task, we make use of recent progress in deep learning models based on neural networks [31]. Despite the fact that neural network models are known since many decades [35–40] recent advances in the learning methodology revived them [31]. Specifically, in contrast to classic neural network models, deep neural networks can have a large number of hidden layers. Each of these layers builds a complex representation of the previous layers as a result from nonlinear transformations [41].

Deep learning models have been successfully used in many application areas, most notably in image recognition [41, 42] and speech recognition [43]. In computational biology, deep learning is still at an early stage and the studied data come mostly from the DNA-level. For instance, alternative splicing and protein binding patterns have been studied [44–46]. For analyzing gene expression data and especially for the classification of cancer very little is know. One of the few studies in this area is from [47]. They used data from DNA microarrays to classify lung adenocarcinoma and squamous cell carcinoma. However, they used not only the classification data set but additional data from further disease stages to train their deep learning model in the unsupervised phase. This is possible since during the unsupervised training phase no labels are needed nor used. A more conventional analysis has been conducted by [30]. They used deep forest models for the classification of various cancer types based on gene expression data from both DNA microarray and RNA-Seq. For this Stacked Denoising Autoencoders in combination with ANNs and SVMs have been used. However, neither of these studies investigated ncRNAs, only coding RNAs have been analyzed.

Our paper is organized as follows. In the next section we present details about the lung cancer data and the methods for our analysis. Then we present our results and a discussion of these. We finish the paper with concluding remarks.

## Methods

### Lung cancer data

For our analysis, we are using RNA-seq data from lung cancer in Koreans [28]. The data were generated with Illumina HiSeq 2000 and contain matched information about 62 subjects with lung adenocarcinoma and 62 subjects from adjacent normal tissues (control samples). These samples are taken from cancer tissues whose driver mutations were not detected by screening tests (Sanger sequencing for EGFR and for KRAS point mutations and fluorescence in situ hybridization for EML4-ALK fusion); see [28] for details. Access to the data is provided via Gene Expression Omnibus (GEO) (http://www.ncbi.nlm.nih.gov/geo/), accession number GSE40419.

The processing pipeline for the data include the following steps [48]: The dataset [28] was extracted from SRA archive [49]. The samples were aligned using Bowtie2 [50], with 1 mismatch to the hg38 human genome [51]. The reads were summarized to count vectors with samExploreR [48, 52] and normalized with RPKM (Reads Per Kilobase Million - also used in [28]). RefSeq annotation was taken for reads mapping procedure. Then transcripts that were not expressed or at a very low intensity were removed. On a technical note, we would like to remark that we repeated our analysis by using TPM (Transcripts Per Kilobase Million) instead of RPKM but found no noticeable differences in our results.

TopHat and STAR would be alternative choices instead of Bowti2, which are quite commonly used in transcriptomics studies. Their major difference from BowTie2 is an opportunity to account for reads in splice-affected regions. However, in [53] it was argued that these reads make no impact on transcript abundance quantification, and therefore, for the purpose of Differential Gene Expression Bowtie2 alignment is an applicable procedure.

Using the human genome annotation databases Reference Sequence (hg38) as reference to map RNA sequencing reads to protein coding RNAs, we find expression levels for 36,742 RefSeq genes. After filtering out redundant entries this results in 22,427 gene transcripts. Removing genes with low expression values (maximum expression smaller than 3 RPKM) or genes with a small standard variation results in 12,360 genes corresponding to protein coding RNAs we used for our analysis.

For a similar analysis for non-coding transcripts we find 3124 non-coding RNAs (ncRNAs) for RPKM and 1398 ncRNAs for TPM (16828 before filtering). That means the majority of ncRNAs is expressed at a very low level. Among these the most important ncRNAs are [54]:

- microRNA: miRNA
- ribosomal RNA: rRNA
- small interfering RNA: siRNA
- long non-coding RNAs: long ncRNAs or lncRNAs
- small nuclear ribonucleic acid: snRNA

### Error measures

For our analysis, we need to assess the performance of a binary classification. The results form such a classification can be summarized by a confusion matrix shown in Table 1.

**Table 1:** Confusion matrix summarizing the results for binary classifications

		True class	
		Positive	Negative
Predicted class	Positive	True positives (TP)	False positives (FP)
	Negative	False negatives (FN)	True negatives (TN)

From the confusion matrix in Table 1 one can derive the following three performance metrics [55, 56].

- Accuracy (*A*) $=\frac {TP+TN}{TP+FP+FN+TN}$
- True positive rate (TPR) (also called sensitivity) $=\frac {TP}{TP+FN}$
- True negative rate (TNR) (also called specificity) $=\frac {TN}{TN+FP}$

For our analysis a true positive (TP) indicates a correctly predicted lung adenocarcinoma sample and a true negative (TN) a correctly predicted control sample. Hence, the true positive rate is with respect to lung adenocarcinoma and the true negative rate for the control samples. The true positive rate (TPR), also called sensitivity, evaluates the proportion of all positives correctly identified and the true negative rate (TNR), also called specificity, evaluates the proportion of all negatives correctly identified. With respect to the confusion matrix the TPR and the TNR are symmetrically defined by exchanging the class labels. Overall, the TPR has a focus on positive labels which correspond in our case to lung adenocarcinoma patients and the TNR has a focus on negative labels corresponding to control patients. In contrast, the accuracy assesses the overall classification performance for both classes in a weighted manner. In addition, we evaluate the area under the receiver operator characteristics (AUROC) curve [57]. For the construction of a ROC curve one needs pairs of TPR and FPR values. These values are obtained for different threshold parameters of the classifier. Practically, it is sufficient to rank all values for the samples and use successively different threshold values. Technical details are described in [55].

Due to the fact that the number of samples in both classes is exactly the same, none of our measures suffers from negative consequences of imbalanced classes [58].

For assessing the variability of our results, we use a 10-fold cross validation (CV) in order to estimate the standard errors of the performance measures. CV splits the data into 10-folds whereby one fold is used for training (estimation of parameters of the models) and 9-folds are used for testing. CV is a resampling method that is the gold standard for error estimations in order to avoid a high bias [59, 60].

### Deep belief networks

For our analysis, we are using Deep Belief Networks (DBNs) [31]. DBN models are trained in two separate phases. In the first phase, a Restricted Boltzmann Machine (RBM) is used to initialize the model, and in the second phase a supervised method is used for tuning of the parameters [61]. These steps are called pre-training phase and fine-tuning phase. For the fine-tuning we are using the *stochastic gradient descent* either in combination with the *basic backpropagation* (Bprop) algorithm or the *resilient backpropagation* (Rprop) algorithm, whereas the *resilient backpropagation* (Rprop) algorithm is a more efficient, faster variant of the Bprop.

In the following, we describe the pre-training and fine-tuning steps briefly.

#### Unsupervised pre-training

In principle, it is possible to learn neural network models solely by supervised learning methods skipping a pre-training step entirely. However, it has been shown that pre-training with a suitable initialization of the model parameters, i.e., the weights of the network, can make the supervised learning step much faster and improve the overall performance [41]. The Restricted Boltzmann Machine (RBM) has been introduced for this pre-training step providing for this an unsupervised initialization of the parameters [31, 62]. This allows the training of deep architectures, i.e., networks with many hidden layers, that can achieve better performances than shallow architectures with only one hidden layer.

Technically, the pre-training step of DBNs consists in stacking RBMs, so that the next RBM in a chain is trained by using the previous hidden layer as its input layer, in order to initialize parameters for each layer. In previous studies this approach has been found to be efficient [63]. Furthermore, this allows the order of the layers to be trained to be chosen freely. For example, one can train the last layer first and after a certain number of epochs the preceding layers can be trained [31]. For our analysis, we are using a Restricted Boltzmann Machine model with binary units and the contrastive divergence (CD) algorithm for approximating the log-likelihood of the RBM.

#### Supervised fine-tuning

In the supervised training step the parameters of the model are optimized by fine-tuning the model. For this step, the class labels of the training data are used, which makes this step supervised. In contrast, the pre-training step does not make use of these labels and for this reason is unsupervised.

The resilient backpropagation (Rprop) algorithm is a motified version of the backpropagation algorithm. The purpose for introdicing this algorithm was to speed-up the backpropagation (Bprop) algorithm [64]. There are several realizations of Rprop available [65]. However, for our study, we are using the iRprop^+^ algorithm. iRprop^+^, as well as all other realizations, are available in the darch package [66]. In addition to iRprop^+^, we are using the basic backpropagation algorithm (Bprop) for reasons of comparison.

#### Network architecture

The architecture of the neural network is a parameter of the model that needs to be defined. From previous studies it is known that there is not one type of network architecture that is best under all conditions but the choice of the architecture is data and problem dependent. For instance, some studies use a decreasing architecture (the number of neurons in the hidden layers decreases) [63], whereas others use an increasing architecture [67] or even a constant architecture [68]. This implies that there is no consensus for deep learning networks about the shape of the architecture. For this reason, we were testing a vast number of different network architectures to find the best one for our problem. In the “Results” section, we provide information about the architectures we were testing.

## Results

In the following, we will analyze RNA-seq data from lung cancer patients in two ways. First, we will only use gene expression values from protein-coding RNAs corresponding to mRNAs. Second, we will only use gene expression values from non-coding RNAs (ncRNAs) including miRNAs.

### Protein-coding genes

The RNA-seq data for our analysis consist of 62 samples from lung adenocarcinoma and 62 samples from adjacent normal tissues corresponding to control samples. For our first analysis will only use gene expression data from protein-coding genes. For our data set this corresponds to 12360 mRNAs (genes). We will use these data for a binary classification separating adenocarcinoma samples from control samples. The results of this classification are summarized in Table 2.

**Table 2:**
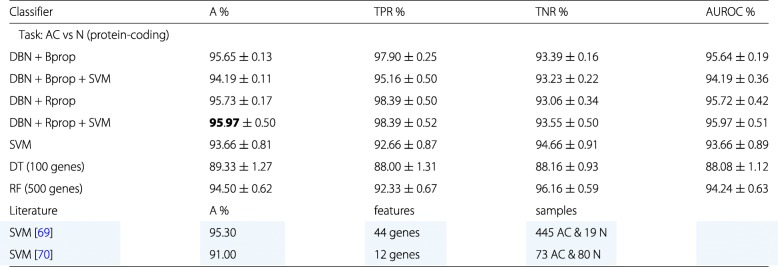
Summary of the best classification results for lung cancer

Only RNA-seq data from protein-coding genes were used. The best classifier is highlighted in green. In addition, for comparison reference results from the literature are shown, highlighted in blue

Our results are interesting for several reasons. First, the DBN classifier in combination with SVM or alone outperform the SVM, decision tree (DT) and random forrest (RF) for almost all combinations. Second, Bprop and Rprop perform similarly with only a slight advantage for Rprop. This is somehow surprising because it is know that Rprop performs in general better than Bprop. Third, the deep learning classifiers outperform the SVM but only slightly.

In Table 3 we show some examples for the further configurations we studied for DBNs and SVMs. The best results are color highlighted. Overall, the architecture of the DBN seems to tolerate a large variability in the number of hidden layers as well as their sizes. This hold for both algorithms Bprop and Rprop. Interestingly, repeating the above analysis by using various feature selection mechanisms we did not find a beneficial effect for the deep learning model, in contrast, the performance decreases. Similar results hold also for the SVM. Only the decision tree and random forrest classifiers benefit somewhat from a feature selection and we obtain for 100 genes the best results for a decision tree and for 500 gene the best results for a random forrest classifier. However, the benefit for both methods is moderate because without feature selection the accuracy of both classifiers drops by only 2.5*%*.

**Table 3:** A: DBN results for the RNA-Seq data set. B: SVM results for the RNA-Seq data set

A.							
		DBN			DBN and SVM		
Model	Architecture	A %	TPR %	TNR %	A %	TPR %	TNR %
DBN + Bprop	A-500-250-100-1	95.48±0.25	98.06±0.40	92.90±0.36	94.13±0.27	97.58±0.27	91.94±0.34
DBN + Bprop	A-100-1	95.65±0.13	97.90±0.25	93.39±0.16	94.19±0.11	95.16±0.50	93.23±0.22
DBN + Rprop	A-5-10-1	95.73±0.17	98.39±0.50	93.06±0.34	95.89±0.08	98.39±0.50	93.39±0.16
DBN + Rprop	A-50-1	95.16±0.50	98.39±0.50	91.94±0.50	**95.97±0.50**	98.39±0.50	93.55±0.50
Task: AC vs N; non-coding, A=3124							
DBN + Bprop	A-2000-1000-500-1	**96.77±0.50**	100±0.50	93.55±0.50	95.16±0.50	96.94±0.16	93.39±0.16
DBN + Bprop	A-100-1	95.97±0.50	100±0.50	91.94±0.50	95.48±0.13	97.42±0.26	93.55±0.50
DBN + Rprop	A-5-10-1	96.05±0.22	98.39±0.50	93.71±0.45	96.45±0.13	98.06±0.22	94.84±0.22
DBN + Rprop	A-50-1	94.35±0.50	100±0.50	88.71±0.50	95.48±0.13	98.39±0.50	92.58±0.26
B.							
		Radial			Linear		
Data	Features	A %	TPR %	TNR %	A %	TPR %	TNR %
Protein-coding	12360	**93.66±0.81**	92.66±0.87	94.66±0.91	93.00±0.89	90.00±0.89	96.00±0.85
Non-coding	3124	91.41±0.97	88.00±0.99	94.83±0.56	**93.91±0.89**	90.50±0.73	97.33±0.72

The best results are shown in bold

In order to compare our results with previous findings, we performed a literature search. From this we found two related studied, the results are shown in Table 3 color highlighted in blue. Specifically, in [69] a SVM was used to classify 445 adenocarcinoma (AC) and 19 normal (N) samples from RNA-seq data using 44 gene features. Also in [70] a SVM was used. In their case, 73 adenocarcinoma (AC) and 80 normal (N) samples from RNA-seq data were classified using 12 gene features. Overall, we observe that our results are competitive and even provide slightly better results.

There are further studies about the classification of lung cancer, but they are less close to our setting. For instance, in [71] lung adenocarcinoma with normal lung tissue samples have been compared. However, the data set they used contained only 5 normal samples in total. This is from a statistical point of view highly problematic because the number of normal samples in the training sets seems far too small. The accuracy values they obtained were 97.2% (SVM), 97.2% (Radial basis function Neural Nets), 97.2% (Multi-layer perceptron), 95.8% (Bayesian network), 94.4% (J48 decision tree) and 95.8% (random forest). These results were obtained for 917 features. By using features selection mechanism reducing the dimension to 50 they were able to further improve the results for some methods. However, also these improvements are only very moderate.

Another study by [72] used 86 lung adenocarcinomas samples and 10 non-neoplastic samples, but no controls. They tested two different methods and achieved for both 100% accuracy using 813 and 9 features respectively. Also the study by [73] did not use normal samples, but compared 150 lung adenocarcinomas samples with 31 malignant pleural mesothelioma for expression data from DNA microarrays. Comparing seven different methods they found accuracy values between 96.13% (decision tree) and 99.45% (SVM). For the decision tree 3 features have been used and for the SVM 12533 (no feature selection has been applied). The same data set has been used in several other studies, e.g., in [74] in comparison with 9 different classifiers and they found a SVM with linear kernel to perform best with an accuracy of 98.13% for 20 feature genes and in [75] with a two-gene classifier (TGC) based on finding optimal cuts they reached 93% with 2 genes as features.

We want to highlight that in addition to the differences mentioned above, all of these less close studies used DNA microarray data but no RNA-seq data.

Regarding the application of deep learning classifiers, in [47] samples from adenocarcinoma and squamous cell carcinoma were compared with various deep learning methods. They obtained accuracy values ranging from 87.5 to 93.33%. The best performing method utilized a PCA for dimension reduction as input for an one or multi-layered sparse autoencoder connected to a SVM (Gaussian kernel) as classifier. It is important to highlight that in addition to the data mentioned, they used further gene expression data from lung cancer patients for their unsupervised learning phase to improve the general learning behavior. This included also lung cancer patients with other tumor types than adenocarcinoma or squamous cell carcinoma. Such data could be used because in the unsupervised phase for learning the autoencoder the labels are ignored. We found only one further study that applied deep learning to gene expression data from DNA microarray [76], but not from lung cancer. However, also in [76] a feature selection mechanism was used (Infinite Feature Selection [77]) selecting 500 genes as input for the deep learning models.

As a result from this literature overview its seems that our study is the first to investigate the classification of deep learning classifiers without feature selection.

### Non-coding RNAs

In this section we are repeating a similar analysis as in the previous section, however, with one important difference. Instead of using RNA-seq data from genes coding for proteins we will use RNA-seq data from genes that do not code for proteins (non-coding genes) leading to non-coding RNAs. In our data set, we have in total 3124 non-coding RNAs.

Our results are summarized in Table 4. This time all configurations of the DBNs outperform the SVM, decision tree and random forrest classifier clearly. Interestingly, learning with Bprop is more beneficial than using Rprop. A possible explanation for this is that the non-coding data set has distinctly less features than the data set for coding RNAs. The difference is about a factor of 4 (3.96=12360/3124). Hence, one can use more complex network architectures that are learnable.

**Table 4:**
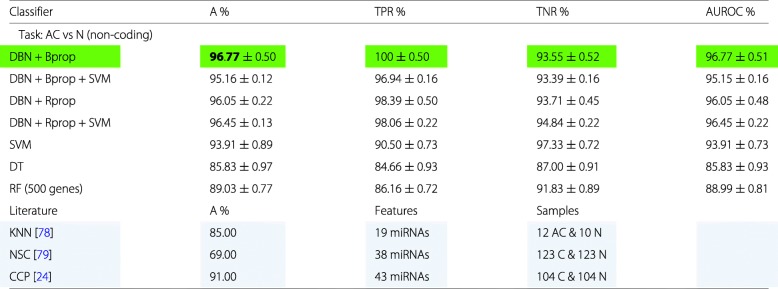
Summary of the best classification results for lung cancer

Only RNA-seq data from non-coding RNAs were used. The best classifier is highlighted in green. In addition, for comparison reference results from the literature are shown, highlighted in blue

In Table 4, we included also three results from the literature (highlighted in blue) that performed a comparable analysis. In [78] a K-nearest neighbors (KNN) classifier was used for 19 miRNAs to classify 12 adenocarcinoma (AC) patients from 10 controls (N). In [79] a nearest shrunken centroids (NSC) classifier has been used for 38 miRNAs. For their analysis they use 123 carcinoma (C) samples and 123 controls (N). The carcinomas class included adenocarcinoma, squamous-cell carcinoma and small-cell carcinoma. Similarly, in [24] a compound covariate predictor (CCP) has been applied to 43 miRNAs found to be differentially expressed. However, as one can see from Table 4 all of their classification results were worse than ours.

Recently, it was shown that information about the presence and absence of miRNA isoforms (isomiRs) can be used to discriminate between 32 cancer subtypes [80]. The average sensitivity of a SVM classifier trained on RNAs-seq data was 90%, and between 80-100% for data sets from diverse platforms (Affimetrix miRNA Array, AVI SOLID sequencing) [81]. Of note, the classifier differentiated between lung adenocarcinoma and lung squamous cell carcinoma [80].

All of the results from the literature have in common that they all used feature selection and they all performed worse than our best results. Also to the best of our knowledge there is no study that included ncRNAs beyond miRNAs in their analysis.

### Comparison and feature selection

**Fig. 1:**
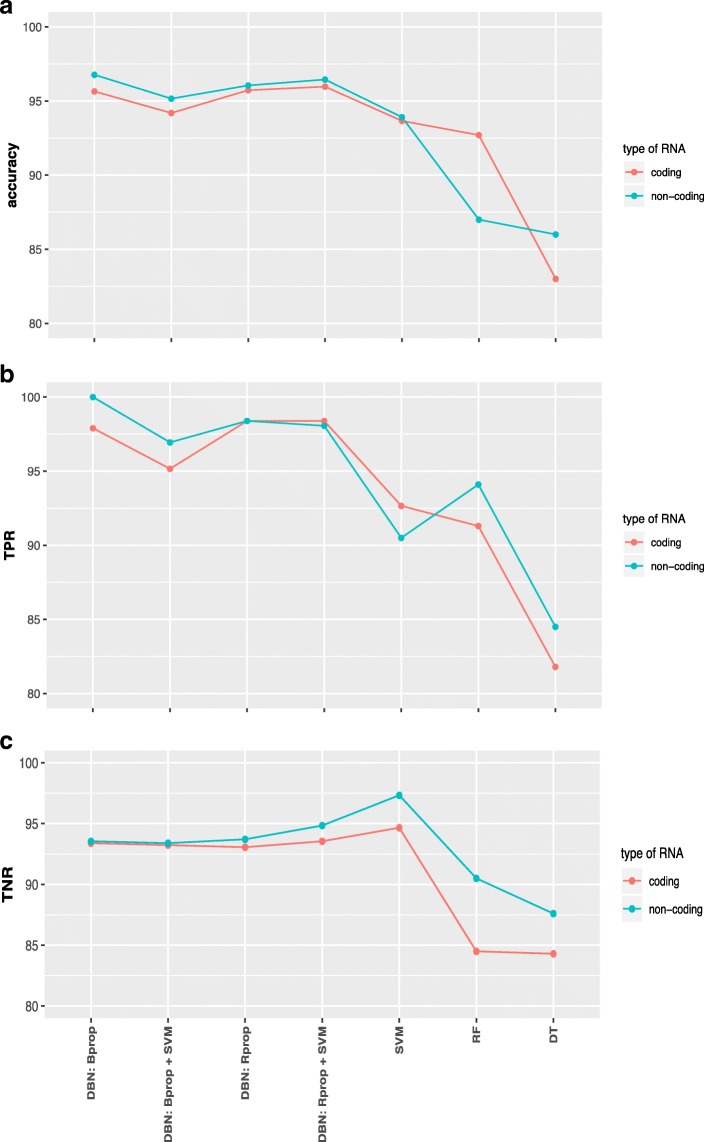
Comparison of the results for different classification methods (see x-axis) and data from coding RNAs (red) and non-coding RNAs (green) for RPKM normalization. **a** Accuracy of the classification. **b** True positive rate. **c** True negative rate

Next, we are comparing the results we obtained for different classification methods (here RF: random forest, DT: decision tree). In Fig. 1a-c we show a summary of results for the coding RNAs (red lines) and non-coding RNAs (green lines) for accuracy, true positive rate and true negative rate.

Overall, the accuracy values for data from non-coding RNAs are for all compared classification methods higher than for data from coding RNAs (Fig. 1a). The differences are not large but sufficient to demonstrate that the predictive abilities of non-coding RNAs are at least as good as for coding RNAs for the diagnostics of lung adenocarcinoma. The fact that this holds independently of the used classification method is an indicator for the robustness of this finding irrespectively of the specific statistical methodology.

Due to the fact that deep learning networks do not require a feature selection mechanisms for reducing the dimension of the input data we did not use such a mechanism as a preprocessing step for our analysis so far. However, now we want to study the effect of feature selection [82, 83] in a systematic way.

**Fig. 2:**
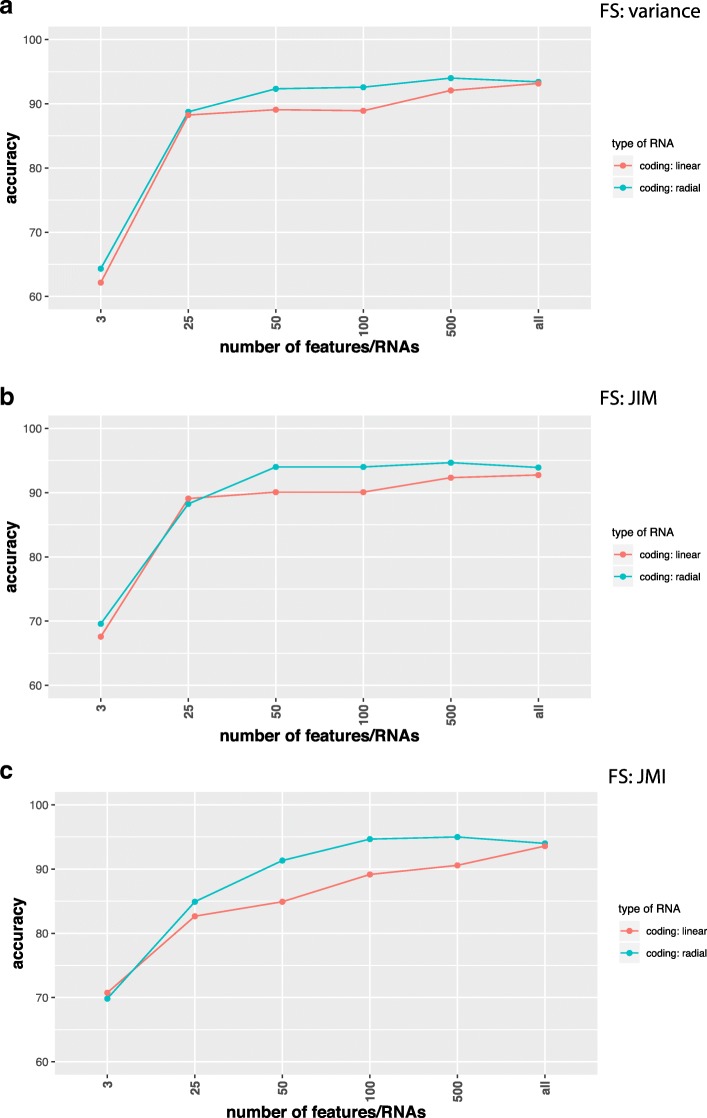
Comparison of classification results for SVMs with a linear (red) and radial (green) basis kernel in dependence on the number of input features/RNAs (x-axis). Data are from coding RNAs for RPKM normalization and the label ’all’ corresponds to 12360 RNAs. Feature selection methods used are **a** Variance, **b** JIM and **c** JMI

**Fig. 3:**
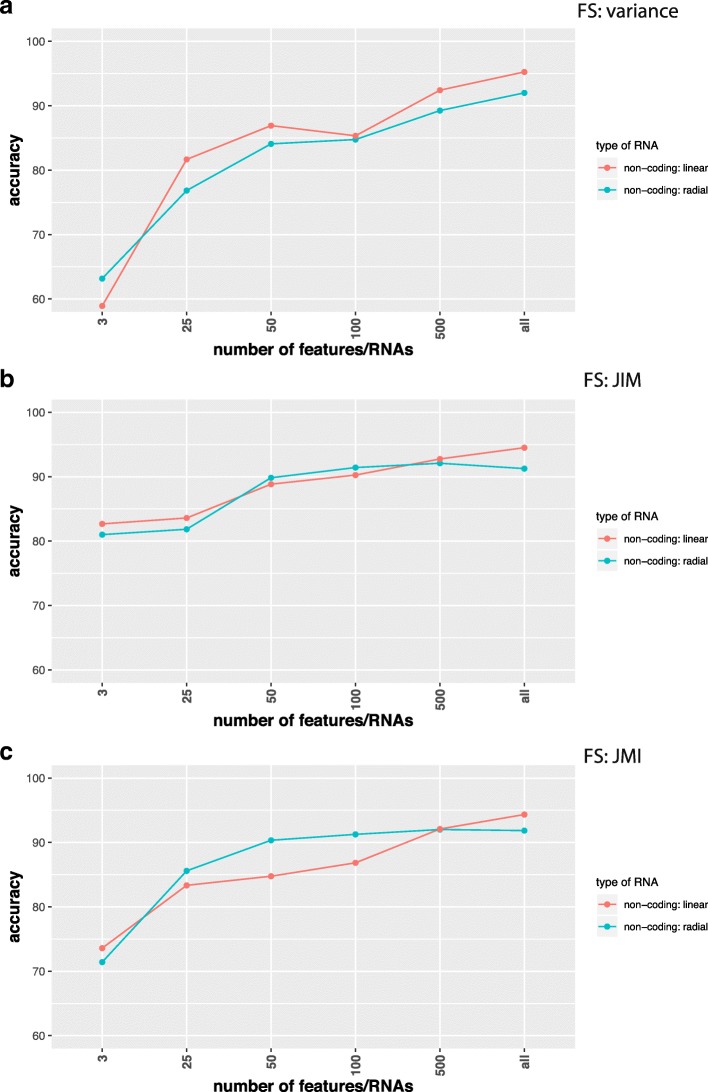
Comparison of classification results for SVMs with a linear (red) and radial (green) basis kernel in dependence on the number of input features/RNAs (x-axis). Data are from non-coding RNAs for RPKM normalization and the label ’all’ corresponds to 3124 RNAs. Feature selection methods used are **a** Variance, **b** JIM and **c** JMI

**Fig. 4:**
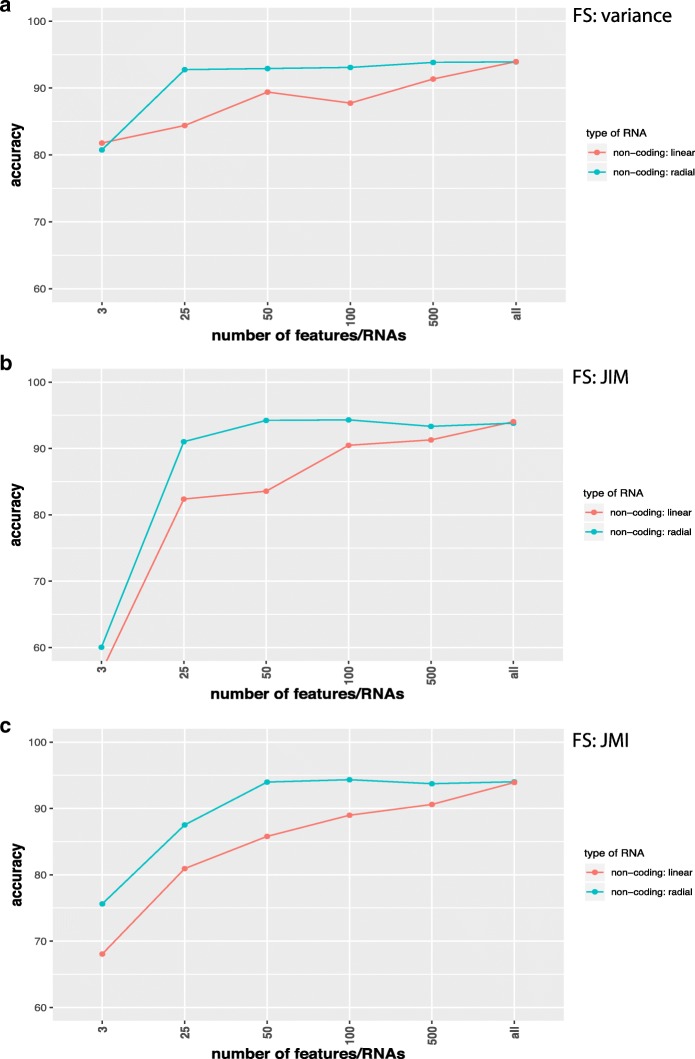
Comparison of classification results for SVMs with a linear (red) and radial (green) basis kernel in dependence on the number of input features/RNAs (x-axis). Data are from non-coding RNAs for TPM normalization and the label ’all’ corresponds to 1398 ncRNAs. Feature selection methods used are **a** Variance, **b** JIM and **c** JMI

**Fig. 5:**
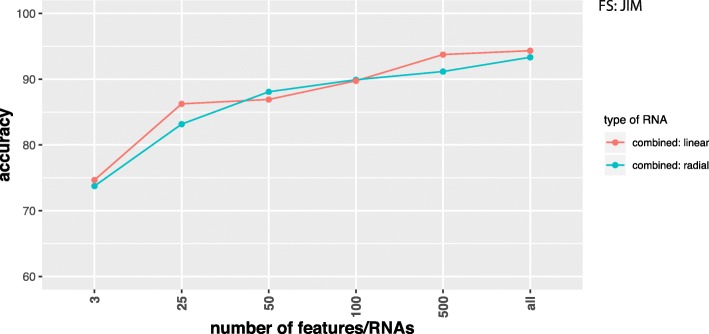
Comparison of classification results for SVMs with a linear (red) and radial (green) basis kernel in dependence on the number of input features/RNAs (x-axis). Data combine coding and non-coding RNAs and the label ’all’ corresponds to 15484 RNAs. Feature selection method used is JIM. Data were RPKM normalized

In Figs. 2, 3, 4, and 5 we show results for the effect of different feature selection mechanisms. Specifically, we used three different feature selection mechanisms producing gene-scores that can be used for rank ordering the genes. We used the variance (A), JIM (joint impurity filter) (B) and JMI (joint mutual information) (C).

The joint impurity filter (JIM) is defined as,

1 $$\begin{array}{@{}rcl@{}} J(X_{j}) = \sum_{W_{k} \in S} G(X_{j}, W_{k}; Y_{j}). \end{array} $$

Here *X*_*j*_ is the expression value of feature *j*, *Y*_*j*_ is its the outcome variable (class label), *S* is the set of already selected features and *G*(*X*_*j*_,*W*_*k*_;*Y*_*j*_) is the Gini impurity gain,

2 $$\begin{array}{@{}rcl@{}} G(X;Y) = \sum_{xy} \frac{p_{xy}^{2}}{p_{x}} - \sum_{y} p_{y}^{2}. \end{array} $$

The method starts with the feature that maximizes the impurity gain and then greedily adds new features that maximize *J*(*X*_*j*_).

The joint mutual information (JMI) is defined as [84],

3 $$\begin{array}{@{}rcl@{}} J(X_{j}) = \sum_{W_{i} \in S} I(X_{j}, W_{i}; Y_{j}). \end{array} $$

Here *S* is again the set of already selected features and *I* is the joint mutual information between *X*_*j*_,*W*_*i*_ and *Y*_*j*_. The method adds new features *X*_*j*_∉*S* in a greedy way by maximizing *J*(*X*_*j*_).

In Fig. 2 we show results for coding RNAs and in Fig. 3 for non-coding RNAs. The label ’all’ corresponds to 12360 coding RNAs (Fig. 2) and 3124 non-coding RNAs (Fig. 3) respectively and FS indicates the applied feature selection mechanism. As one can see, regardless of the chosen feature selection mechanism or the kernel of the SVM reducing the number of features/RNAs is not beneficial for the performance of the classification. In Fig. 5 we show also results for combined data, i.e., we used the coding and non-coding RNAs together. In this case ’all’ corresponds to 15484 RNAs. Also for this, no benefit is gained from reducing the number of features (only results for JIM are shown). All of these results have been obtained for RPKM normalized data. In order to demonstrate that the normalization has no effect on our results we show in Fig. 4 results for TPM normalized data. Results for other feature selection mechanisms are similar (not shown).

Overall, these investigations demonstrate that a feature selection does not have a positive effect on the classifiers.

## Discussion

From analyzing the classification of lung adenocarcinoma patients by using a number of different state-of-the-art classification methods, we found that data from non-coding RNAs have a comparable classification performance as data from coding RNAs, whereas for DBN we found an even better performance. This demonstrates that (I) both data sources (coding and non-coding RNAs) contain a comparable amount of information regarding the underlying disease and (II) the results are robust and do not depend on a particular classification method. From this we conclude the equivalence of predictive information as captured by non-coding RNAs and protein coding RNAs and their utility for computational diagnostics tasks in lung adenocarcinoma.

An intuitive biological explanation for this observation is given by the idea underlying the epigenetic landscape [85] or the model for transcription regulation by [86]. In both studies it has been realized that molecular mechanisms are organized by networks and these are containing feedback loops [87–89] connecting all system variables. Despite the fact that ncRNAs do not code for proteins, their activity is concerted alongside ordinary cell activities and, hence, their expression levels reflect ordinary cell functioning. What is more interesting is the fact that the signal captured by the ncRNAs is equally strong for diagnostic purposes as of coding RNAs.

In our study, we showed that for the classification of lung adenocarcinoma patients a feature selection is not beneficial, but reduces the prediction accuracy for the DNN and SVM (see Figs. 2, 3, 4, and 5). In contrast, the best classifications were obtained without using feature selection. We demonstrated this for different feature selection methods. In this respect, we want to clarify the difference between a feature filtering and a feature selection. A filtering of features is used to remove variables (in our case either mRNAs or ncRNAs) from the analysis that do not carry any information. For instance, for biological cells it is know that not all genes are expressed in all cell types. For this reason, the corresponding mRNAs are not present in such cell types and any measured counts are due to pure noise of the high-throughput device. In order to remove such features we filtered inactive RNAs. In contrast, a feature selection operates on *valid* features, which are all supposed to carry information, and select from these a small subset to which the analysis is limited. Even for the data sets we analyzed without using any feature selection mechanism we applied a feature filtering to remove noise from the data.

For general deep learning networks one could state that these models perform also feature selection, but in an implicit manner. This is certainly true but the key point is this is part of the model itself and, hence, the feature selection and the classifier are merged with each other. Furthermore, such *higher order* features, corresponding to representations in deeper layers, are non-linear combinations of the original input features and for this reason they lost their biological interpretability. In other words, even if one would dissect such low dimensional features from the model, they would no longer correspond to a few genes or RNAs because information from all of these would be present to a certain extend. For SVMs such an implicit feature selection mechanism is less obvious.

The RNA world population is constantly expanding, e.g., new types of ncRNAs with tissue-, disease-, sex-, population origin- and race- specific expression rates were recently described [90]. These miRNAs isoform (isomiRs) were able to discriminate between 32 TCGA cancer types, probably because of the high expression specificity. It is well understood by now that ncRNAs are key regulators of physiological programs in developmental and disease states and are particularly relevant in cancer, identified as oncogenic drivers and tumor supressors for all major cancer types [91]. ncRNAs link associated genes into regulatory networks, as well as regulate each other [92]. Therefore it is plausible that ncRNAs have at least the same predictive capabilities as mRNAs.

To the best of our knowledge, we are the first to use ncRNAs beyond miRNAs for the computational classification of cancer. All previous studies limited their focus on miRNAs when classifying disease stages, e.g., [24, 78, 79]. Furthermore, we are also the first to perform a direct comparison of the classification capabilities of coding and non-coding RNAs.

Despite the popularity of general deep learning methods in the last years in many fields [41, 42], the analysis of gene expression data is so far understudied. Our results show that different variations of deep belief networks, using either Bprop or Rprop for the fine-tuning phase, lead to competitive results compared to SVMs. Also the combination of deep belief networks with SVMs is fruitful, which even showed for the coding RNAs the best results. These results are encouraging and demonstrate that, at least for large data sets, as used in our study, deep learning classifiers are capable of dealing with gene expression data.

Finally, we think it is important to emphasize that our analysis was only possible due to the general capability of DBNs and LIBSVMs [33] to deal efficiently with high-dimensional input data as a result from omitting feature selection mechanisms. This is certainly not the case for every classification method.

## Conclusion

In our study, we assessed the entire information content of coding RNAs (mRNAs) and non-coding RNAs provided in RNA-seq data by studying the data with and without feature selection. Overall, from analyzing a large-scale data set from lung adenocarcinoma patients we found:

1. For the diagnostic classification, ncRNAs have as much predictive abilities as coding RNAs. These results hold for different state-of-the-art classification methods, including deep learning methods (deep belief networks), SVMs and combinations.
  ⇒ This may point to a new application area for ncRNAs in the computational diagnostics of lung cancer and potentially other disorders.
2. Feature selection reduces this predictive ability in both cases and the best prediction accuracy is obtained without feature selection.
  ⇒ This eliminates a general problem all biomarker studies suffering from, which is the definition of the optimal biomarker set [93].
3. Deep belief networks perform competitively to SVMs.
  ⇒ Despite this positive finding, compared to the overwhelming success of DBN for image analysis, the result differences are not large enough to claim a dominating performance.

From reviewing the literature it seems our study is the first to use ncRNAs beyond miRNAs for the computational classification of cancer. All previous studies limited their focus on miRNAs when classifying disease stages, e.g., [24, 78, 79]. Furthermore, we are also the first to perform a direct comparison of the classification capabilities of protein coding RNAs and non-coding RNAs.

For future studies, it would be interesting to expand our analysis to other cancer types and general complex disorders. It would be interesting to see if also other diseases exhibit the same behavior as we observed for lung cancer. We expect similar results to hold for other cancers but for general complex disorders predictions are more difficult.

In summary, our investigations underline the importance of general ncRNAs in understanding the complex etiology of lung cancer and suggest to conduct similar studies for other cancer types and possibly other complex disorders.

## Data Availability

Access to the data is provided via Gene Expression Omnibus (GEO) (http://www.ncbi.nlm.nih.gov/geo/), accession number GSE40419.
